# Higher-Order Aberrations in Myopic Eyes

**Published:** 2010-01

**Authors:** Farid Karimian, Sepehr Feizi, Azade Doozande

**Affiliations:** Labbafinejad Medical Center, Shahid Beheshti University of Medical Sciences, Tehran, Iran

**Keywords:** Higher-Order Aberrations, Myopia, Zywave Aberrometer, Hartmann-Shack Aberrometer

## Abstract

**Purpose:**

To evaluate the correlation between refractive error and higher-order aberrations (HOAs) in patients with myopic astigmatism.

**Methods:**

HOAs were measured using the Zywave II aberrometer over a 6 mm pupil. Correlations between HOAs and myopia, astigmatism, and age were analyzed.

**Results:**

One hundred and twenty-six eyes of 63 subjects with mean age of 26.4±5.9 years were studied. Mean spherical equivalent refractive error and refractive astigmatism were −4.94±1.63 D and 0.96±1.06 D, respectively. The most common higher-order aberration was primary horizontal trefoil with mean value of 0.069±0.152 μm followed by spherical aberration (−0.064±0.130 μm) and primary vertical coma (−0.038±0.148 μm). As the order of aberration increased from third to fifth, its contribution to total HOA decreased: 53.9% for third order, 31.9% for fourth order, and 14.2% for fifth order aberrations. Significant correlations were observed between spherical equivalent refractive error and primary horizontal coma (R=0.231, P=0.022), and root mean square (RMS) of spherical aberration (R=0.213, P=0.031); between astigmatism and RMS of total HOA (R=0.251, P=0.032), RMS of fourth order aberration (R=0.35, P<0.001), and primary horizontal coma (R=0.314, P=0.004). Spherical aberration (R=0.214, P=0.034) and secondary vertical coma (R=0.203, P=0.031) significantly increased with age.

**Conclusion:**

Primary horizontal trefoil, spherical aberration and primary vertical coma are the predominant higher-order aberrations in eyes with myopic astigmatism.

## INTRODUCTION

Higher-order aberrations (HOAs) are small optical irregularities or imperfections of the eye which cannot be corrected by simple sphere and cylinder corrections. Many authors believe that HOAs are the reason many patients complain of halo, glare and decreased contrast sensitivity after successful corneal refractive surgery.^1^,^2^ In the normal eye, 90% of total aberrations are caused by the cornea. New diagnostic technologies enable the detection and correction of ocular aberrations beyond defocus and astigmatism^3^,^4^ by applying the root mean square (RMS) of Zernike coefficient polynomials.^5^,^6^

The purpose of this study was to measure and evaluate the distribution of HOAs in myopic eyes and to determine any correlation between the degree of refractive error (myopia and astigmatism) and HOAs.

## METHODS

The study included young refractive surgery candidates with a completely normal ocular examination except for myopic refractive error. Exclusion criteria were history of ocular or corneal surgery or trauma, corneal scar, lens or media opacity, pathologic myopia or severe chorioretinal atrophy which could alter vision and wavefront measurements, and best spectacle-corrected visual acuity (BSCVA) of 20/40 or worse. Soft and rigid gas-permeable hard contact lenses were discontinued for at least 2 and 6 weeks, respectively and measurements were taken provided that there was no corneal warpage.

Measurement of HOAs and wavefront analysis were performed across a 6.0 mm pupil using Zywave II aberrometer with Zywave software version 5.2 (Bausch & Lomb, Rochester, NY, USA) in a dark room. The Zywave II aberrometer is a Hartmann-Shack wavefront sensor applying light in the near infrared range (λ=785 nm).^7^ In this aberrometer, the pupil is sampled through a square array of lenslets with a fixed pitch, the number of spots (samples) depends on the chosen pupil diameter. Each measurement consists of five sequential runs; the system computes the average of three best compatible measurements after rejecting the two measurements with higher deviations from the mean.

All measurements were performed by one experienced technician using the same machine and procedure. If the natural scotopic pupil failed to reach 6.0 mm, it was dilated with 2.5% phenylephrine eye drops as recommended by the manufacturer. Aberrometric measurements were performed approximately 30 minutes after instillation of the drop. To avoid instrument accommodation, the eye was fogged approximately 1.00 D during measurements.^8^

Zernike polynomials up to the fifth order were used for data analysis. All Zernike coefficients were transformed to the standard form as recommended by the Optical Society of America.^9^ Analyzed parameters included Zernike coefficients from third to fifth orders; RMS of total HOAs from third to fifth orders; RMS of fourth order spherical aberration (square root of the sum of squared coefficients of Z_4_^0^); RMS of coma-like aberration (square root of the sum of squared coefficients of Z_3_^−1^, Z_3_^1^, Z_5_^−1^, and Z_5_^1^); RMS of trefoil-like aberrations (square root of the sum of squared coefficients of Z_3_^−3^, Z_3_^3^, Z_5_^−3^, and Z_5_^3^); and RMS of third, fourth and fifth order aberrations. Correlations between HOAs and myopia, astigmatism and age were examined using multiple linear regression analysis and Pearson’s correlation coefficient (R) with significance level set at 0.05.

## RESULTS

One hundred and twenty-six eyes of 63 myopic patients including 41 (65%) female and 22 (35%) male subjects with mean age of 26.4±5.9 (range, 18–43) years met the study criteria. Mean spherical equivalent refractive error was −4.94±1.63 (range, −1.13 to −8.50) D and mean astigmatism was 0.96±1.06 (range, 0 to 4.50) D; BSCVA was 20/20 or better in all eyes.

From the mean of total aberrations (RMS) 53.9% were in the third; 31.9% in the fourth; and 14.2% in the fifth orders of aberration (Table 1). Considering the Zernike coefficient of each HOA, primary horizontal trefoil (Z_3_^3^) had the highest mean followed by spherical aberration (Z_4_^0^) and primary vertical coma (Z_3_^−1^) (Table 2).

**Figure 1:**
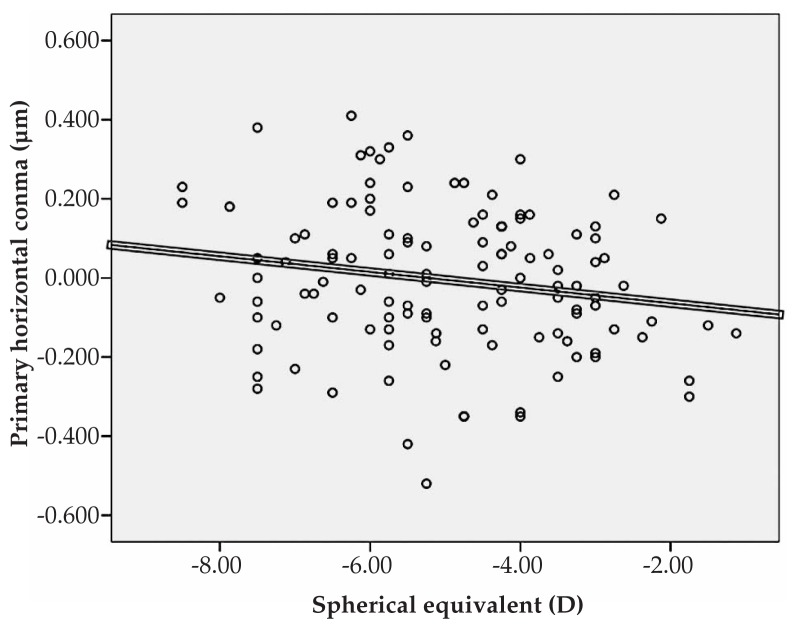
Correlation between spherical equivalent refractive error and primary horizontal coma (R=0.231, P=0.022).

**Figure 2:**
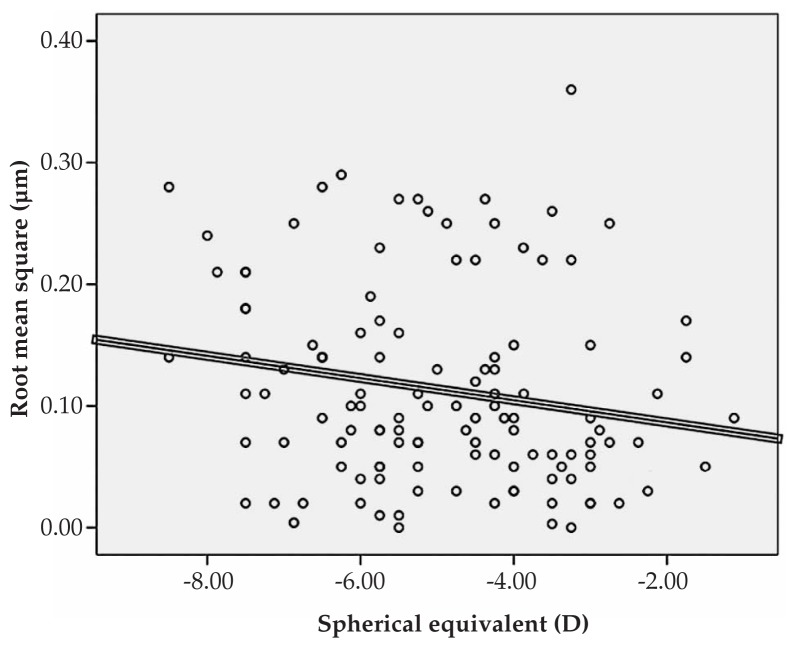
Significant correlation between spherical equivalent refractive error and root mean square of spherical aberration (R=0.213, P=0.031).

**Figure 3:**
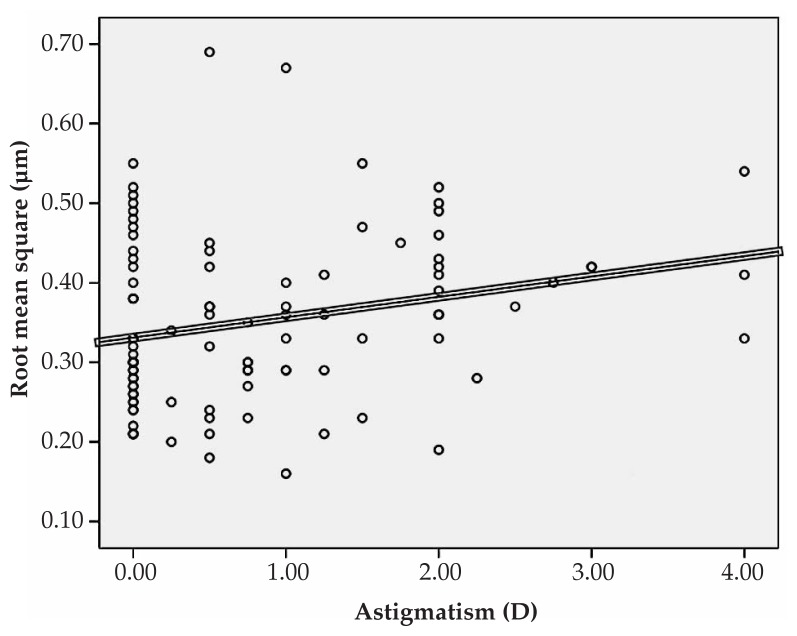
Significant correlation between astigmatism and root mean square of total higher-order aberrations (R=0.251, P=0.032).

**Figure 4:**
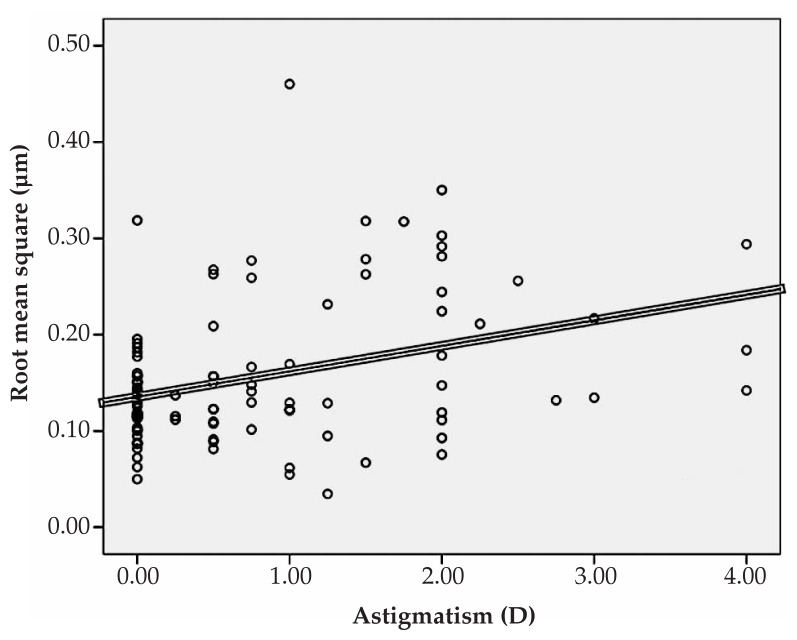
Significant correlation between astigmatism and root mean square of fourth order aberrations (R=0.35, P<0.001).

**Figure 5:**
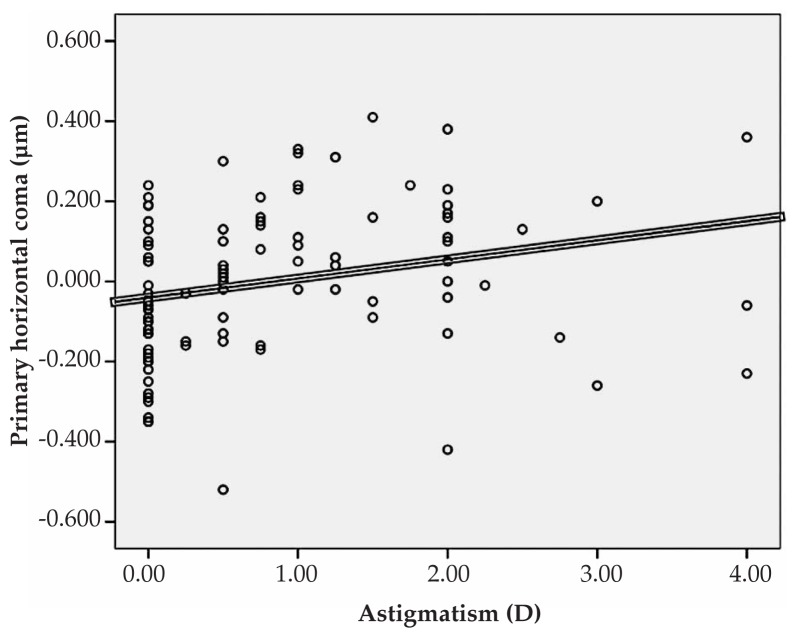
Significant correlation between astigmatism and Zernike coefficient of primary horizontal coma (R=0.314, P=0.004).

**Figure 6:**
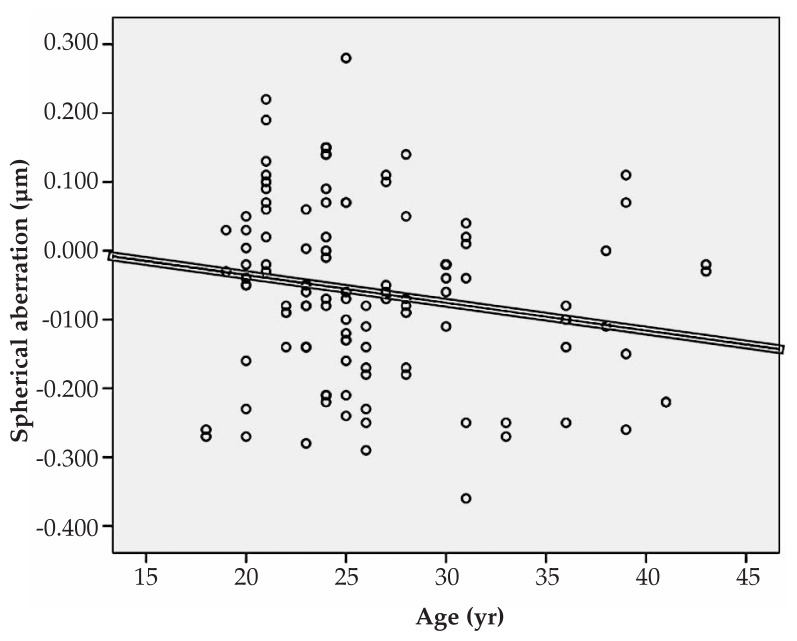
Significant correlation between age and spherical aberration (R=0.214, P=0.034).

**Figure 7:**
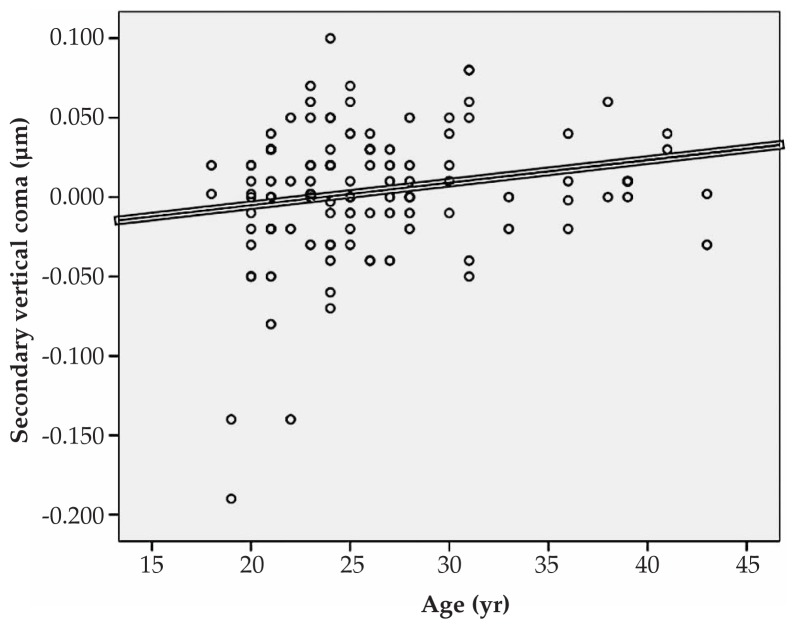
Significant correlation between age and secondary vertical coma (R=0.203, P=0.031).

Multiple linear regression analysis revealed significant correlations between spherical equivalent refractive error and primary horizontal coma (R=0.231, P=0.022), and the RMS of spherical aberration (R=0.213, P=0.031) (Figures 1, 2). No significant correlation was found between spherical equivalent refractive error and the RMS of total HOAs or the RMS of any order of aberrations. A significant direct correlation was also observed between astigmatism and the RMS of total HOA (R=0.251, P=0.032) and the RMS of fourth order aberrations (R=0.35, P<0.001). Considering individual Zernike polynomials, there was a significant correlation between astigmatism and primary horizontal coma (R=0.314, P=0.004) (Figures 3, 4, 5). Furthermore it was noted that spherical aberration (R=0.214, P=0.034) and secondary vertical coma (R=0.203, P=0.031) increased significantly with age (Figures 6, 7).

## DISCUSSION

This study explores changes in higher-order aberrations as a function of refractive error (spherical equivalent and astigmatism) and age. Multiple linear regression analysis showed that spherical equivalent refractive error was significantly correlated with primary horizontal coma and the RMS of spherical aberration. These findings are in good accordance with results reported by Applegate^10^ who found dramatically increased coma and spherical aberrations in myopic eyes using a subjective single-pass aberroscope. Similarly using a Shack-Hartmann aberrometer, Paquin et al^11^ found that optical quality was worse in myopic eyes and that high amounts of coma were more frequent in high myopia. Using a subjective ray-tracing technique, He et al^12^ measured aberrations in 146 young adults and found that myopic eyes have slightly higher combined fourth order and higher aberrations as compared to emmetropic eyes. But similar to the current study, they failed to find a significant correlation between total aberrations and spherical equivalent refractive error. Wei et al^13^ showed that there was no correlation between the degree of myopia and the RMS of total higher order aberrations or third to fifth order RMS. Analyzing individual Zernike coefficients rather than RMS values, they found a slightly significant correlation between myopia and primary horizontal trefoil.

Despite these findings, the correlation between refractive error and HOAs remains a matter of controversy. Collins et al,^14^ using an objective double-pass aberroscope, reported lower average spherical aberrations in high myopes than emmetropes. Cheng et al^15^ also concluded that wavefront aberrations were unrelated to refractive error in a population of 200 normal eyes. Maybe, such conflicting conclusions in the aforementioned studies can be attributed to high variability in monochromatic aberrations in myopic eyes. Alternatively, it may be due to lack of a standard method for measurement and interpretation of HOAs. Further studies with larger sample size utilizing a systematic approach are necessary to address this issue.

In concordance with previous reports,^16^–^18^ we noted that the contribution of average RMS of higher order aberrations decreased as the order increased: third order aberrations predominated, followed by fourth and fifth order aberrations. Wang et al^16^ investigated HOAs from third to sixth orders using WaveScan System across a 6.0 mm pupil in 532 eyes with mean WaveScan spherical equivalent of −3.39±2.84 (range, −11.56 to +7.60) D and found that spherical aberration was the predominant aberration followed by primary vertical coma. In contrast, we observed that primary horizontal trefoil had the highest mean followed by spherical aberration and primary vertical coma. This difference may be due to the range of refractive errors evaluated in each study; we analyzed HOAs only among myopic eyes while in the aforesaid study, both myopic and hyperopic subjects were evaluated.

We found positive correlations between the amount of astigmatism and the RMS of HOA, the RMS of fourth order aberration, and primary horizontal coma. But, there was no association between astigmatism and vertical coma, vertical trefoil, horizontal trefoil, and spherical aberrations. Other investigators have reported the influence of astigmatism on wavefront aberrations. Slight but significant correlations between astigmatism and primary horizontal coma, and between astigmatism and primary horizontal trefoil were reported by Wei et al.^13^ Furthermore, Cheng et al^15^ reported slightly larger total higher-order RMS in astigmatic eyes which supports our findings. Zheng et al^19^ conducted a study on 226 eyes of 113 patients and evaluated the influence of the amount and axis of astigmatism on HOAs. To evaluate the pure effect of astigmatism on contrast sensitivity function (CSF) and aberration, the investigators only corrected the spherical component of refractive errors and left the astigmatic component uncorrected. By dividing the patients into three groups based on amount of astigmatism, they found that increasing astigmatism was associated with increasing coma aberrations, secondary coma aberrations, third order, fifth order, and total HOAs. However, the fourth order aberration remained constant.

The current study demonstrated statistically significant correlations between age and spherical aberration, and secondary vertical coma which is consistent with previous reports.^16^,^20^ Such associations may be due to changes in the cornea or crystalline lens which occur with aging. For example, corneal astigmatism usually shifts from with-the-rule to against-the-rule over time. Furthermore, the crystalline lens starts to demonstrate alterations in refractive index and therefore, changes in aberrations occur due to cataract formation.^21^–^24^ Since we measured optical aberrations of the eye as a whole system, we cannot attribute the observed increase in spherical aberration and secondary vertical coma to changes in the cornea or the crystalline lens caused by lenticular astigmatism or aging. Other studies^25^–^27^ have reported no correlation between aging and corneal spherical aberration, which implies that the increasing spherical aberration with age is caused by lenticular rather than corneal changes. Furthermore, age range was limited in our study (18 to 43 years), thereby our observations regarding the effect of age on HOAs may not be applicable to other age groups. Further evaluations, particularly longitudinal studies, are required to determine how much alterations in ocular aberrations are attributable to age related changes in the cornea.

Applegate et al^28^ reported that for an equal amount of RMS error, different coefficients of Zernike polynomials affect visual function to varying degrees. They concluded that aberrations close to the center of the Zernike table (e.g., coma, spherical aberration, secondary astigmatism) cause greater distortion of vision than those located at the periphery of the table. In addition, we demonstrated that the contribution of each higher-order aberration to total HOAs decreases with increasing order. Based on these two observations, one may conclude that lower order and more central aberrations, affect image quality to a greater extent. Therefore, lower-order aberrations including defocus (sphere) and astigmatism remain the most significant contributors to optical system quality and constitute the top priority for correction during refractive surgery. The next priority would be to deal with higher-order aberrations located higher in the Zernike table such as primary coma and those involving central vision like spherical aberration. Later, one can proceed to more inferiorly and peripherally located HOAs. In other words, correcting HOAs without completely eliminating lower-order aberrations may not improve visual performance and meet patients’ expectations.

In summary, we found primary horizontal trefoil to be the predominant HOA in a young myopic population with spherical equivalent refractive error ranging from −1.13 to −8.5 D. Spherical and coma-like aberrations are HOAs associated with increasing myopia and age. Astigmatism is significantly correlated with total HOAs, fourth order and coma-like aberrations.

## Figures and Tables

**Table 1:** Root mean square (RMS) values for different aberrations

Aberration	RMS	

	Mean±SD (μm)	Range (μm)
Total	6.85±2.4	2.50–12.78
Total HOA	0.35±0.12	0.13–1.02
Spherical	0.12±0.08	0.0–0.36
Coma	0.21±0.11	0.03–0.57
Trefoil	0.19±0.1	0.03–0.76
Third order	0.29±0.12	0.10–0.94
Fourth order	0.17±0.08	0.03–0.46
Fifth order	0.08±0.05	0.02–0.41

SD, standard deviation; HOA, high-order aberration

**Table 2:** Coefficient values for each Zernike term from third to fifth order

Zernike Term	Mean±SD (μm)	Range (μm)
Z_3_^−3^	0.0197±0.129	−0.400 to 0.330
Z_3_^−1^	−0.0381±0.148	−0.380 to 0.440
Z_3_^1^	−0.0067±0.180	−0.520 to 0.410
Z_3_^3^	0.0690±0.152	−0.420 to 0.640
Z_4_^−4^	0.0124±0.052	−0.110 to 0.170
Z_4_^−2^	0.0017±0.061	−0.250 to 0.240
Z_4_^0^	−0.0642±0.130	−0.360 to 0.280
Z_4_^2^	0.0066±0.066	−0.170 to 0.180
Z_4_^4^	−0.0028±0.053	−0.170 to 0.120
Z_5_^−5^	0.0054±0.038	−0.120 to 0.090
Z_5_^−3^	0.0015±0.033	−0.060 to 0.130
Z_5_^−1^	0.0035±0.043	−0.190 to 0.100
Z_5_^1^	0.0049±0.028	−0.060 to 0.070
Z_5_^3^	0.0014±0.025	−0.100 to 0.060
Z_5_^5^	−0.0041±0.049	−0.400 to 0.100

SD, standard deviation
